# ESCO2 promotes lung adenocarcinoma progression by regulating hnRNPA1 acetylation

**DOI:** 10.1186/s13046-021-01858-1

**Published:** 2021-02-11

**Authors:** Hui-er Zhu, Tao Li, Shengnan Shi, De-xiong Chen, Weiping Chen, Hui Chen

**Affiliations:** 1 Department of General Practice, The Third Affiliated Hospital of Guangzhou Medical University, Guangzhou Medical University, Guangzhou, Guangdong 510150 P.R. China; 2 Department of Anesthesiology, The Third Affiliated Hospital of Guangzhou Medical University, Guangzhou Medical University, Guangzhou, Guangdong 510150 P.R. China; 3 State Key Laboratory of Animal Breeding, Institute of Animal Science, Guangdong Academy of Agricultural Sciences, South China Key Laboratory of Animal Nutrition and Feed, Ministry of Agriculture, Guangzhou, 510640 P. R. China; 4 Department of Respiratory, The People’s Hospital of Qingyuan, Sixth Affiliate Hospital of Guangzhou Medical University, Qingyuan, 511518 P. R. China; 5 Department of Pathology, The Third Affiliated Hospital of Guangzhou Medical University, Guangzhou Medical University, Guangzhou, Guangdong 510150 P.R. China

**Keywords:** Acetylation, Metabolism reprogramming, Lung adenocarcinoma, ESCO2, hnRNPA1

## Abstract

**Background:**

Emerging evidence indicates that metabolism reprogramming and abnormal acetylation modification play an important role in lung adenocarcinoma (LUAD) progression, although the mechanism is largely unknown.

**Methods:**

Here, we used three public databases (Oncomine, Gene Expression Omnibus [GEO], The Cancer Genome Atlas [TCGA]) to analyze *ESCO2* (establishment of cohesion 1 homolog 2) expression in LUAD. The biological function of ESCO2 was studiedusing cell proliferation, colony formation, cell migration, and invasion assays in vitro, and mouse xenograft models in vivo. ESCO2 interacting proteins were searched using gene set enrichment analysis (GSEA) and mass spectrometry. Pyruvate kinase M1/2 (*PKM*) mRNA splicing assay was performed using RT-PCR together with restriction digestion. LUAD cell metabolism was studied using glucose uptake assays and lactate production. *ESCO2* expression was significantly upregulated in LUAD tissues, and higher *ESCO2* expression indicated worse prognosis for patients with LUAD.

**Results:**

We found that ESCO2 promoted LUAD cell proliferation and metastasis metabolic reprogramming in vitro and in vivo. Mechanistically, ESCO2 increased hnRNPA1 (heterogeneous nuclear ribonucleoprotein A1) binding to the intronic sequences flanking exon 9 (EI9) of *PKM* mRNA by inhibiting hnRNPA1 nuclear translocation, eventually inhibiting PKM1 isoform formation and inducing PKM2 isoform formation.

**Conclusions:**

Our findings confirm that ESCO2 is a key factor in promoting LUAD malignant progression and suggest that it is a new target for treating LUAD.

**Supplementary Information:**

The online version contains supplementary material available at 10.1186/s13046-021-01858-1.

## Background

Lung cancer is a heterogeneous tumor with high morbidity and mortality, and is a serious threat to human health [1, 2]. Lung adenocarcinoma (LUAD) is the most common histologic type of lung cancer, accounting for about 50% of all lung cancers. On average, more than 7500 people die from cancer every day [3]. More than 35% of all cancer deaths are from lung cancer [4]. In recent years, many targeted therapies, such as anaplastic lymphoma kinase (ALK) [5], EGFR [6], ROS1 [7], RET [8], HER2 [9], and MEK [10], have become available for advanced lung cancer, and more are in development [11]. Although there are many means of treating lung cancer, no specific drugs have been found so far [12]. Due to tumor heterogeneity, there is an urgent need to identify new therapeutic targets.

Establishment of cohesion 1 homolog 2 (ESCO2) is an evolutionarily conserved cohesion acetyltransferase that exerts essential functions in the establishment of sister chromatid cohesion [13]. In recent years, ESCO2 has been identified as an essential factor in cancer progression in multiple human cancers [14–17]. Compared with conventional chemotherapy, ESCO2 may become a more promising option for renal cell carcinoma treatment intervention [15]. *ESCO2* is highly expressed in aggressive melanomas and breast cancer [16, 18]. In gastric cancer, ESCO2 promotes cell proliferation by modulating the p53 and mammalian target of rapamycin (mTOR) signaling pathways [19]. However, its biological function and clinical significance in lung cancer remain unclear.

Heterogeneous nuclear ribonucleoprotein A1 (hnRNPA1) is an RNA-binding protein that associates with pre-mRNAs in the nucleus and influences pre-mRNA processing, as well as other aspects of mRNA metabolism and transport, and plays a key role in regulating alternative splicing [20, 21]. The pyruvate kinaseisoform PKM2 is widely expressed in cancer for maintaining glycolysis-dominant energy metabolism, while PKM1 promotes oxidative phosphorylation [22]. These two isoforms result from mutually exclusive alternative splicing of *PKM* pre-mRNA, reflecting the inclusion of either exon 9 (PKM1) or exon 10 (PKM2), required for tumor cell proliferation [23]. Clinical cancer samples support the notion that hnRNPA1 overexpression decreases the PKM1/PKM2 ratio, which has a positive effect on glycolysis-dominant metabolism [24].

In the present research, we found that high ESCO2 expression in LUAD was associated with poor prognosis. Overexpression of *ESCO2* promoted LUAD cell proliferation, colony formation, migration, and invasion in vitro, while *ESCO2* knockdown inhibited LUAD cell malignant progression in vitro and tumorigenesis and metastasis in vivo*.* Coimmunoprecipitation (Co-IP) and mass spectrometry (MS) analysis suggested that ESCO2 could interact with hnRNPA1, which is involved in mRNA splicing or processing. Moreover, we found that ESCO2 can acetylate hnRNPA1 at lysine 277 (K277) to retain hnRNPA1 in the nucleus. Only in the nucleus can hnRNPA1 regulate PKM splicing to promote PKM2 generation and inhibit PKM1 generation, leading to LUAD metabolism reprogramming. The present study indicates the functional roles of ESCO2 in LUAD progression and that ESCO2 may be a potential therapeutic target for LUAD.

## Materials and methods

### Tissue samples and cell culture

Primary cancer tissue and normal lung tissue were collected from patients with lung cancer at the SixthAffiliated Hospital of Guangzhou Medical University. The cases were collected based on a clear pathological diagnosis and patient consent, and the study was approved by the Internal Review and Science Committee of the Sixth Affiliated Hospital of Guangzhou Medical University. The human LUAD cell lines A549 and NCI-H1975 were purchased from the Cell Bank of the Chinese Academy of Sciences (Shanghai, China) and maintained in RPMI 1640 medium supplemented with 10% fetal bovine serum (FBS). HEK293T cells were purchased from ATCC and cultured in Dulbecco’s modified Eagle’s medium (DMEM) containing 10% FBS. All cells were maintained at 37 °C and5% CO_2_ in a humidified incubator.

### Public database analysis and gene set enrichment analysis (GSEA)

LUAD gene expression datasets of Garber et al., Hou et al. and Okamaya et al. were analyzed via Oncomine database (https://www.oncomine.org). LUAD gene expression datasets (GSE74706, GSE21933, GSE32863, GSE50081 and 31,210) were downloaded from the Gene Expression Omnibus (http://www.ncbi.nlm.nih.gov/geo) database. 515 LUAD and 59 normal lung tissue samples were obtained from The Cancer Genome Atlas (TCGA) dataset (https://portal.gdc.cancer.gov/). The TCGA LUADsamples were subdivided into high and low ESCO2expression groups and analyzed with GSEA 2.0.9 software (http://www.broadinstitute.org/gsea/).

### Plasmid constructs, transfection, and stable silencing

Plasmids were constructed by homologous recombination. Briefly, after primer design and synthesis, complementary DNA (cDNA) was amplified using Phanta Max Super-Fidelity DNA Polymerase (Vazyme, cat: C505). The PCR products were purified and recovered according to the protocol of a general DNA purification and recovery kit (Tiangen Biochemical Technology, DP214–03). Then, recombination, transformation, coating, cloning identification, and plasmid extraction were performed. The mutants of hnRNP A1-HA were produced usingMut Express II Fast Mutagenesis Kit V2 (vazyme, C214). The A549 and NCI-H1975 cells were transfected with overexpression vector using Lipofectamine 2000 (Thermo Fisher Scientific, MA, USA). LUAD cell lines with stable *ESCO2* silencing were constructed using lentivirus pLV3short hairpin RNA (shRNA) as previously described [4]. The lentivirus pLV3-*ESCO2* shRNA was purchased from GenePharma (Shanghai, China). The sequences of the primers and shRNAs used in the study are listed in Supplementary Table S1.

### Immunofluorescence staining

NCI-H1975 cells were transfected with *ESCO2*-FLAG vectors, and plated on glass coverslips. The cell density was about 50%; the cells were rinsed with phosphate-buffered saline (PBS) twice, fixed with 1 mL 4% paraformaldehyde at room temperature for 20 min, and permeabilized with 0.1% Triton X-100 for 7 min. The cells were rinsed twice with precooled PBS and blocked with 2% BSA (bovine serum albumin) at room temperature for 2 h. Primary antibody was added and incubated at 4 °C overnight, following which the samples were incubated with secondary antibodies. The nuclei were stained with DAPI, and examined under a microscope.

### Quantitative real-time PCR (RT-qPCR)

Total RNA was extracted from treated A549 and NCI-H1975 cells using TRIzol total RNA isolation reagent (Invitrogen). Then, cDNA was synthesized from the total RNA using a PrimeScript RT Reagent Kit (TAKARA). *ESCO2* mRNA expression was detected using quantitative PCR (q-PCR) following the manufacturer’s protocol. *ESCO2* and *GAPDH* expression levels were measured using the comparative threshold cycle (2-ΔΔCt) method. The primer sequences used are listed in Supplementary Table S1.

### Western blotting

Proteins were extracted from cells or tissue using lysis buffer (1 mM EDTA, 1% SDS, 5 mM DTT, 10 mM PMSF, 50 mMTris–HCl [pH 8.0], protease inhibitor cocktail). Protein concentrations were determined using the bicinchoninic acid (BCA) assay. Total cell lysates were fractionated by 8% or 10% sodium dodecyl sulfate–polyacrylamide gel electrophoresis (SDS-PAGE), transferred to PVDF membranes. The primary antibodies used are listed in Supplementary Table S1.

### Cell growth and colony formation assays

For the cell growth assay, 1 × 10^4^ treated LUAD cells were seeded in 24-well plates, and counted at 24, 48, 72, 96, and 120 h. For the colony formation assays, 5 × 102 treated LUAD cells were seeded in 6-well plates and cultured in RPMI 1640 medium containing 10% FBS for 8 or 10 days. The clones were fixed in methanol and stained with crystal violet solution.

### Migration and invasion assays

The in vitro migration and invasion assays were performed using Transwell chambers. For the migration assay, 1 × 10^5^ LUAD cells with *ESCO2* overexpression or silencing were cultured in RPMI 1640 medium in the upper compartment of a Transwell chamber. For the invasion assay, 2 × 10^5^ LUAD cells with *ESCO2* overexpression were resuspended in RPMI 1640 medium with 0.1% FBS in Matrigel-coated upper Transwell chambers. For both assays, the bottom chambers were filled with RPMI 1640 medium containing 10% FBS. The chambers were stained with 0.5% crystal violet. Migrated and invaded cells were counted under a microscope.

### In vivo xenograft tumor model

All animal procedures were approved by the Institutional Animal Care and Use Committee of the ThirdAffiliated Hospital of Guangzhou Medical University. For the in vivo tumor growth assay, 5 × 10^6^ control or *ESCO2* knockdown NCI-H1975 cells were injected into the left and right flanks of BALB/c null mice (*n* = 6). After 21 days, all tumors were stripped and weighed. For the in vivo metastasis assay, control or *ESCO2* knockdown NCI-H1975 cells were luciferase (Luc)-labeled using the lentivirus system. NCI-H1975-Luc-NC (negative control) or NCI-H1975-Luc *ESCO2*shRNA cells (2 × 10^6^ cells) were injected into the tail veins of NOD-SCID (nonobese diabetic/severe combined immunodeficient) mice. After 45 days, the metastatic foci were detected using the IVIS 200 imaging system (Xenogen, Alameda, CA, USA).

### Silver staining and mass spectrometry (MS)

HEK293T cells were transfected with *Flag-ESCO2* vector for 48 h using Lipofectamine 2000. Treated HEK293T cells were lysed in Co-IP lysis buffer (P0013, Beyotime, Shanghai, China). Co-IP was performed using anti-FLAG/anti-HAantibodiesand protein A/G agarose beads (sc-2003, Santa Cruz) to extractthe complexes. Gel bands were detected using a silver staining kit (P0017S, Beyotime) combined with MS following the manufacturer’s protocol. According to a previously published method [25], the peptides of the bands were analyzed using nano-LC–MS/MS. The mass spectrometry proteomics data have been deposited to the ProteomeXchange Consortium via the PRIDE partner repository with the dataset identifier PXD023527 and PXD23600.

### In vitro acetylation assay

FLAG-tagged ESCO2, HA-tagged hnRNPA1, and their mutant proteins were purified from HEK293T cells using a FLAG Immunoprecipitation Kit (FLAGIPT1, Sigma) or Anti-HA Immunoprecipitation Kit (IP0010, Sigma). Recombinant ESCO2 proteins were incubated with recombinant hnRNPA1 or its mutants in 30 μL reaction buffer (50 mMNaCl, 50 mMTris-HCl [pH 8.0], 4 mM MgCl_2_, 1 mM DTT, 0.1 mM EDTA, 10% glycerol) at 37 °C for 30 min.

### RNA affinity purification

First, pretreatment streptomycin beads:100 μL streptavidin-agarose beads were rinsed twice using pre-cooled 500 μLbinding buffer (pH 7.5), centrifuged at 4 °C at 2500 rpm for 5 min, and the supernatant was discarded. Then, 1 nmol biotin probe–labeled RNA fragments were bound with 100 μL streptavidin-agarose beads at 4 °C overnight. Cellular nuclear protein was prepared using a Nuclear and Cytoplasmic Extraction Kit (Beyotime). Purified protein or nucleoprotein and tRNA were added to the beads, incubated at 30 °C for 10 min, centrifuged at 4 °C at 2500 rpm for 5 min, and the supernatant was discarded. Then, the pretreatment protein was added, incubated at 4 °C for 2 h, centrifuged at 2500 rpm for 5 min at 4 °C, rinsed twice with pre-cooled binding buffer, and centrifuged at 2500 rpm for 5 min at 4 °C. Finally, the elucidated mixtures were detected using western blotting. The 5′ biotin-labeled RNAs used in the study are listed in Supplementary Table S1.

### RT-PCR and PKM splicing assays

*PKM* splicing assays were performed according to a previous study [26]. Briefly, total mRNA was extracted from cells or tissue samples using TRIzol. mRNA reverse transcription was performed using the PrimeScript RT Reagent Kit (TAKARA). The PCR products were digested using *Pst*I, and the digested mixtures were resolved by 8% non-denaturing PAGE. The primers used are listed in Supplementary Table S1.

### Measurement of glucose uptake and lactate production

A549 and NCI-H1975 cells at the logarithmic growth stage were inoculated in a 12-well plate at 1 × 10^5^ cells/well. The experiment was divided into the control group (non-transfected cells) and transfection group. After 36 h, the cells were incubated with phenol red–free RPMI 1640 medium for 8 h, and the glucose content in the culture supernatant was detected using Glucose Colorimetric Assay kit (BioVision, K606–100) according to the operating instructions. The glucose content of the non-transfected group was used as the control.

Treated LUAD cells were seeded into 6-well plates. Lactate production was measured using a Lactate Colorimetric Assay Kit II (BioVision, K627–100) according to the manufacturer’s protocol. Briefly, at 36 h post-transfection, phenol red–free RPMI 1640 medium without FBS was added to a 6-well plate of subconfluent cells and cultured for 4 h. A standard curve of nmol/well versus the OD450nm (optical density at 450 nm) value was plotted according to the measurement of the lactate standard. The OD450nm values of the sample were applied to the standard curve to calculate the lactate concentrations of the test samples (*n* = 3).

### Statistical analysis

Data are presented as the mean ± standard deviation (SD); data analysis was performed using GraphPad Prism 5. Survival curves were described using Kaplan–Meier plots and were calculated using the log-rank test. Statistical differences between two groups were analyzed using an independent Student’s *t*-test (2-tailed). *P* < 0.05, *p* < 0.01, and *p* < 0.001 were considered statistically significant.

## Results

### ESCO2 is upregulated andassociated with poor prognosis in LUAD

**Fig. 1:**
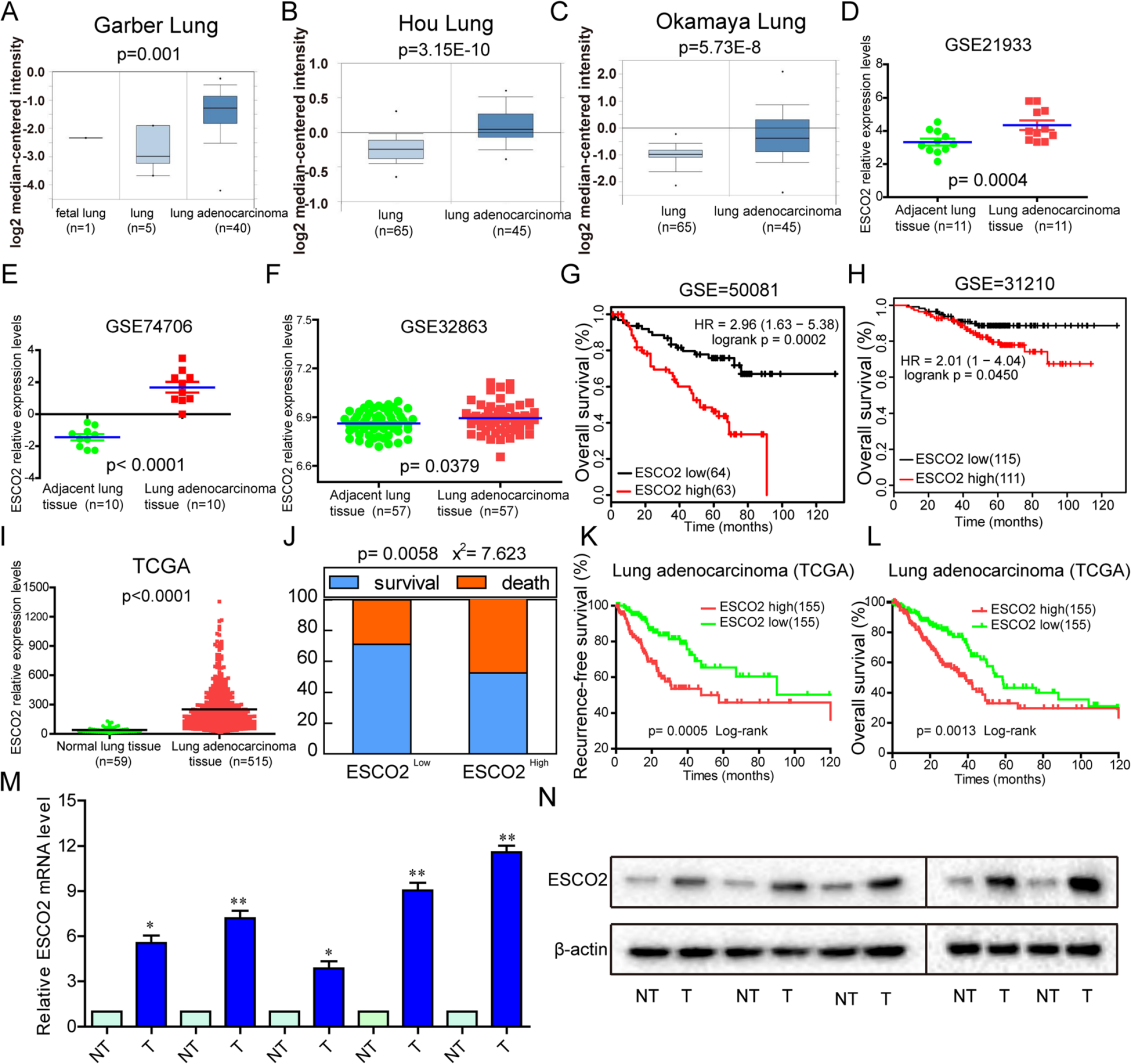
Abnormal expression of ESCO2 is associated with poor prognoses in LUAD. **a**–**c** *ESCO2* mRNA expression in normal lung tissues and LUAD tissues was detected using the Oncomine database. **d**–**f** *ESCO2* mRNA expression in normal lung tissues and paired LUAD tissues was analyzed based on three LUAD mRNA expression profiling datasets (GSE21933, GSE10072, GSE32863). **g** and **h** Patients in the GSE50081 (**g)** and GSE31210 (**h**) datasets with high *ESCO2* expression had shorter OS. (I) *ESCO2* mRNA expression levels were analyzed in normal lung tissues and LUAD tissues from TCGA database. **j**–**l** Analysis of TCGA LUAD cohort showed that, compared with patients with low *ESCO2* expression levels (the lowest 30%), patients with high *ESCO2* mRNA expression (the highest 30%) had higher death rates, shorter disease-free survival, and shorter OS. **m** and **n** *ESCO2* mRNA (**m**) and ESCO2 protein (**n**) expression levels were detected in normal lung tissues and paired LUAD tissues (*n* = 5)

*ESCO2* mRNA expression in normal lung tissues and LUAD tissues (Garbar lung, Hou lung, Okamaya lung) was detected using the Oncomine database. We found that *ESCO2* expression was significantly higher in LUAD tissues than in normal tissues (*p* = 0.001, *p* = 3.15E-10 and *p* = 5.73E-8; Fig. 1a–c). Three *LUAD* mRNA expression profiling datasets (GSE21933, GSE10072, GSE32863) were analyzed for the *ESCO2* mRNA expression levels between LUAD tissues and their adjacent normal tissues. The analysis showed that *ESCO2* expression was significantly upregulated in LUAD tissues compared with the adjacent normal lung tissues (*p* = 0.0004, *p* < 0.0001 and *p* = 0.0379; Fig. 1d–f).

To elucidate the relevance of *ESCO2* overexpression to the survival of patients with LUAD, two publicly accessible microarray datasets of patients with LUAD were analyzed. From the GSE50081 and GSE31210 datasets, patients with high *ESCO2* expression had shorter overall survival (OS) (*p* = 0.0002 and *p* = 0.0450; Fig. 1g and h). Moreover, The Cancer Genome Atlas (TCGA) database showed that *ESCO2* gene expression levels in LUAD tissue was significantly higher than that in normal lung tissue (*p* < 0.0001; Fig. 1i). TCGA LUAD cohort showed that, compared with patients with low *ESCO2* expression (the lowest 30%), patients with high *ESCO2* mRNA expression (the highest 30%) had higher rates of death, shorter disease-free survival, and shorter OS (*p* = 0.0058, *p* = 0.0005 and *p* = 0.0013; Fig. 1j–l). We performed a chi-square test to clarify the correlation between *ESCO2* low and high expression on the clinicopathological features of LUAD. Our findings indicated that high *ESCO2* mRNA expression levels werecorrelated significantly positively with pT status (*p* = 0.001), pN status (*p* = 0.003), and clinical stage (*p* = 0.005) (Table 1). To demonstrate *ESCO2* expression in LUAD tissues, we performed qPCR and western blotting on five LUAD tissues and the corresponding non-tumor (NT) tissue samples; the results showed that ESCO2 mRNA and protein expression levels werelower in the NT tissues than in the LUAD tissues (Fig. 1m and n). Collectively, our findings indicate that ESCO2 level was significantly upregulated in LUAD and was significantly negatively correlated with OS and DFS, suggesting that ESCO2 may serve as a molecular marker for LUAD treatment and as a promoter of tumorigenesis.

**Table 1:** Comparison of clinical features between LUAD patients with low and high ESCO2 levels in TCGA database

Clinical character^#^	Clinical groups	ESCO2		x^2^	*p* value
		High (***n*** = 155) (%)	-Low (***n*** = 155) (%)		
Age (years)	≤ 60	54 (34.8)	41 (26.5)	2.611	0.106
	> 60	95 (61.3)	108 (69.7)		
Gender***	Male	91 (58.7)	59 (38.1)	13.23	< 0.001
	Female	64 (41.3)	96 (61.9)		
ALK Translocation	No	54 (34.8)	71 (45.8)	0.377	0.539
	Yes	12 (7.7)	12 (7.7)		
pT status***	T1 T2 ~ T4	37 (23.9)	66 (42.6)	12.27	< 0.001
		117 (75.5)	88 (56.8)		
pN status**	N0	88 (56.8)	110 (71.0)	8.771	0.003
	N1 ~ N2	66 (42.6)	40 (25.8)		
pM status*	M0	106 (68.4)	101 (65.2)	4.026	0.045
	M1	13 (8.4)	4 (2.6)		
Recurred/Progressed**	No	62 (40.0)	88 (56.8)	8.317	0.004
	Yes	67 (43.2)	46 (29.7)		
Clinical Stage**	Stage I ~ IIA	83 (53.5)	106 (68.4)	7.761	0.005
	Stage IIB ~ IV	70 (45.2)	46 (29.7)		

Differences with *p<0.05, **p<0.01 or ***p<0.001 were considered statistically significant

#American Joint Committee on Cancer classification (Version 7) (AJCC)

### ESCO2 overexpression promotes the malignant phenotype of LUAD cells

**Fig. 2:**
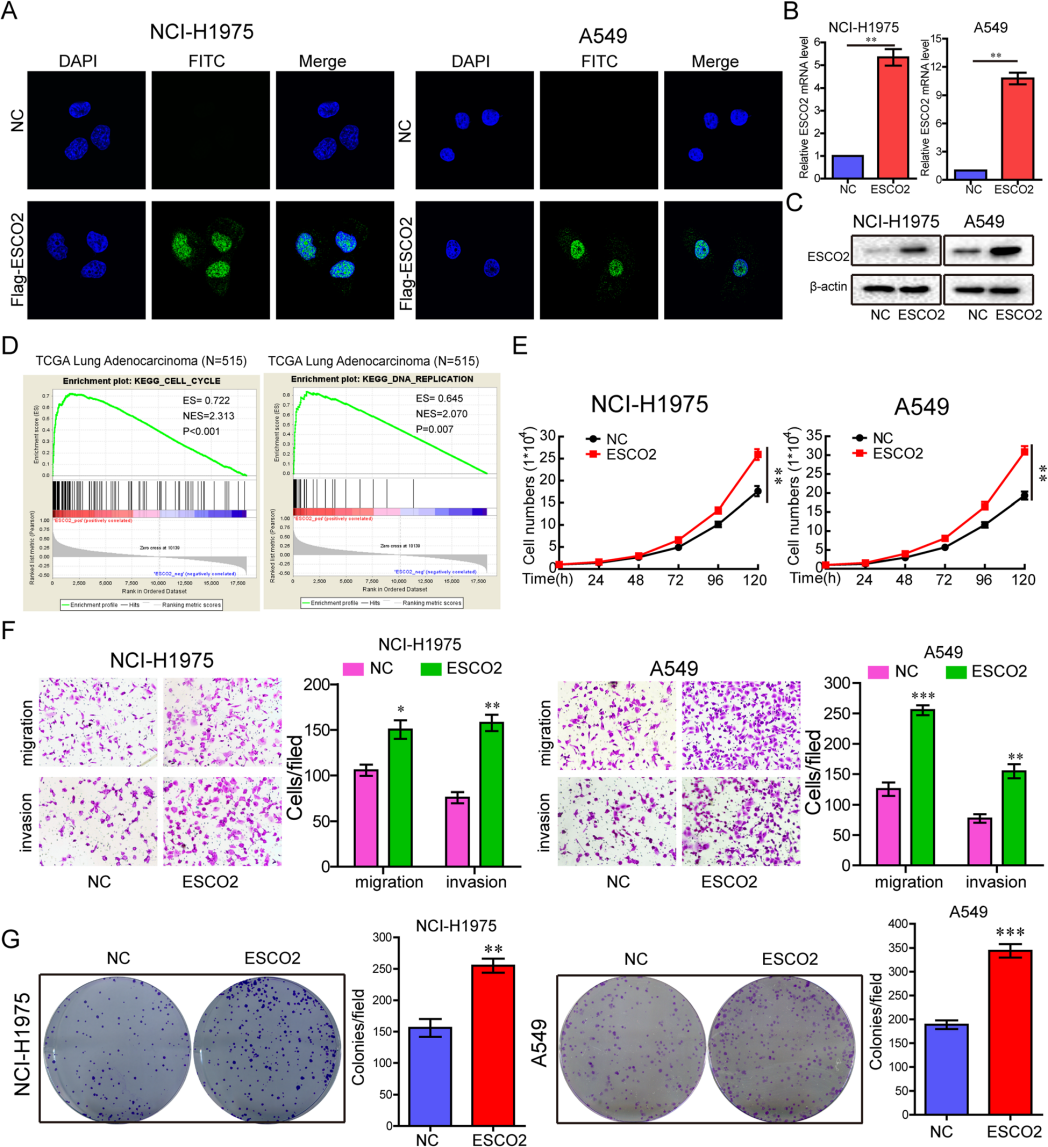
Enforced expression of *ESCO2* promotes LUAD cell growth, metastasis, and colony formation in vitro. **a**–**c** *ESCO2-*FLAG constructs were transfected into NCI-H1975 and A549 cells; FLAG was immunostained using anti-FLAG antibodies (**a**); ESCO2 mRNA and protein expression levels were detected by q-PCR (**b**) and western blotting (**c**), respectively. **d** GSEA showed that the cell cycle signatures and DNA replication signatures had a significant positive correlation with the *ESCO2* mRNA expression levels in TCGA LUAD cohort. **e**–**g** The effects of *ESCO2* overexpression on cell growth (**e**), migration and invasion (**f**), and colony formation (**g**) were detected in NCI-H1975 and A549 cells. Data are the mean ± SD. *p < 0.05, **p < 0.01, ***p < 0.001

To demonstrate the influence of ESCO2 on cancer growth, metastasis, and colony formation, we generated a*ESCO2-*FLAG construct, where the FLAG tag (six amino acids) was fused to the full-length *ESCO2* transcript. The constructs were transfected into NCI-H1975 and A549 cells, and the expression of the construct was examined using immunofluorescence staining, RT-qPCR, and western blotting. The results suggested the successful transfection of *ESCO2* into the cells (Fig. 2a–c).

The global mRNA expression profiles of TCGA LUAD were detected using gene set enrichment analysis (GSEA) software. GSEA plots showed that the cell cycle signatures (*p* < 0.001) and DNA replication signatures (*p* = 0.007) had a significantly positive correlation with *ESCO2* mRNA expression levels in TCGA LUAD cohort (Fig. 2d). To identify the effects of *ESCO2* overexpression in the malignant phenotype of LUAD cells, the *ESCO2* construct was transiently transfected into NCI-H1975 and A549 cells. *ESCO2* overexpression significantly promoted cell growth in both NCI-H1975 and A549 cells (*p* = 0.0011 and *p* = 0.0012; Fig. 2e) and significantly increased cell invasion and migration (Fig. 2f). Colony formation was also significantly increased significantly in the *ESCO2* overexpression LUAD cells (*p* = 0.0054 and *p* = 0.0051; Fig. 2g).

### Stable silencing of ESCO2 inhibited the aggressive phenotype of LUAD cells in vitro and in vivo

**Fig. 3:**
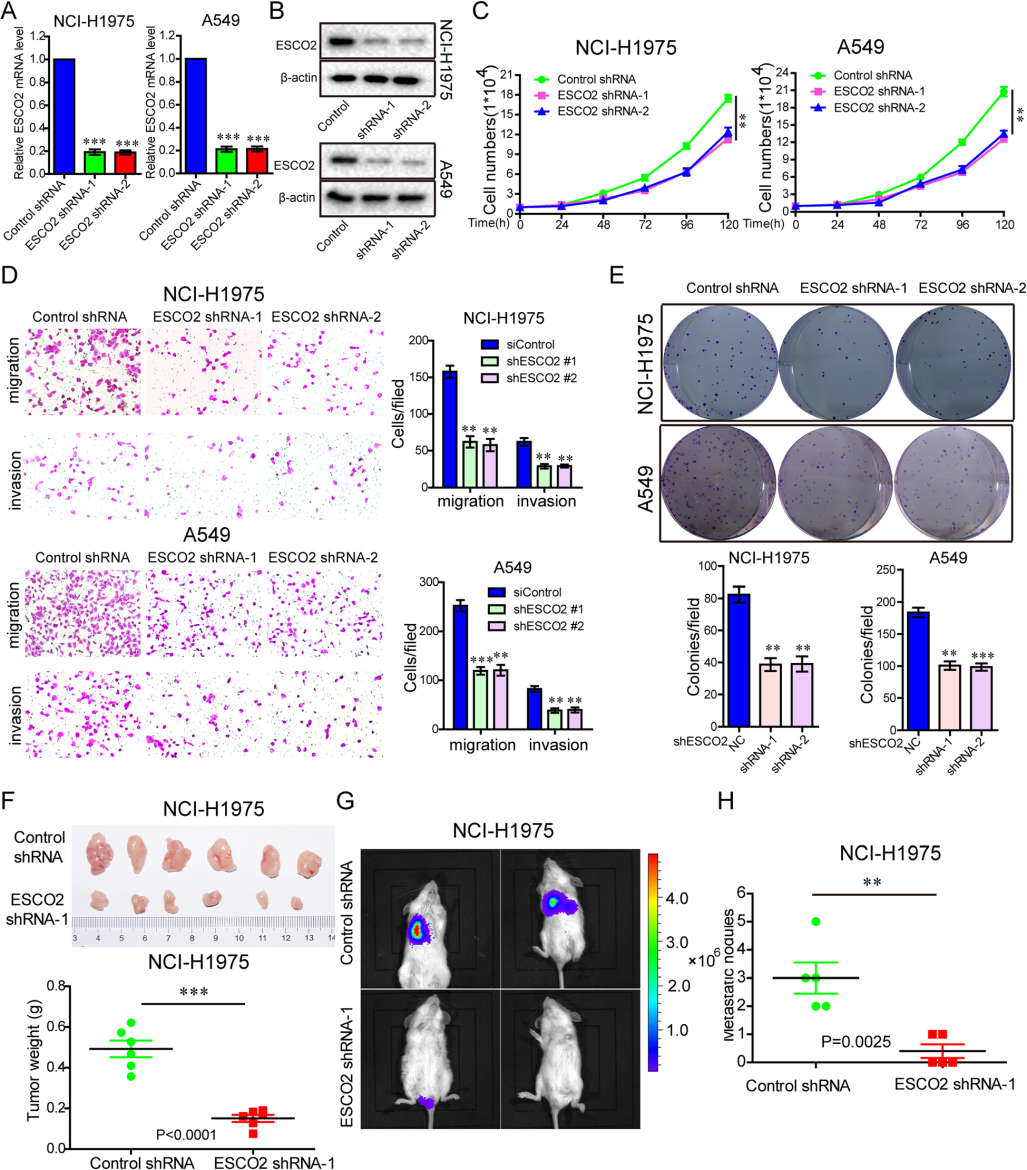
Stable silencing of *ESCO2* expression inhibits the malignant phenotype of LUAD cells in vitro, and growth and metastasis in vivo*.* **a** and **b** *ESCO2* expression was stably silenced using shRNA; ESCO2 mRNA and protein expression levels in NCI-H1975 and A549 cells were detected by q-PCR (**a**) and western blotting (**b**), respectively. **c**–**e** The effects of *ESCO2* stable silencing on LUAD cell growth (**c**), migration and invasion (**d**), and colony formation (**e**) were detected. **f** The in vivo growth of NCI-H1975 cell lines with *ESCO2* stable silencing was detected (*n* = 6). **g** NOD-SCID mice (*n* = 5) were transplanted with Luc-labeled NCI-H1975 cells (2 × 10^6^ cells/mouse) with *ESCO2* stable silencing to detect in vivo metastasis ability. **h** The lung metastatic nodule number was analyzed. Data are the mean ± SD. *p < 0.05, **p < 0.01, ***p < 0.001

To prove the influence of ESCO2 on the aggressive phenotypeof LUAD cells in vitro, and on growth and metastasis in vivo, *ESCO2* expression was stably silenced using shRNA. ESCO2 mRNA and protein expression levels were detected by RT-qPCR and western blotting, respectively. Comparing with the control shRNA group, the ESCO2 shRNA-1(*p* < 0.001) and ESCO2 shRNA-2 (*p* < 0.001) groups had significantly decreased ESCO2 mRNA and protein levels (Fig. 3a). Comparing with the control shRNA group, the ESCO2 shRNA-1and ESCO2 shRNA-2 groups had alsoobviouslydecreased ESCO2 protein expression (Fig. 3b). *ESCO2* silencing significantly inhibited cell growth (Fig. 3c), migration and invasion (Fig. 3d), and colony formation (Fig. 3e) in both the NCI-H1975 and A549 cells.

In addition, we observed that, contrary to the control shRNA, NCI-H1975 cells with *ESCO2* stable silencing had lower tumor volumes and significantly decreased tumor weights (*p* < 0.0001; Fig. 3f). *ESCO2* stable silencing also suppressed metastasis ability in vivo (Fig. 3g). At the same time, analysis of the lung metastatic nodule number showed that the ESCO2 shRNA-1 group had significantly decreased metastatic nodules (*p* = 0.0025; Fig. 3h).

### ESCO2 acetylated hnRNPA1 at K277

**Fig. 4:**
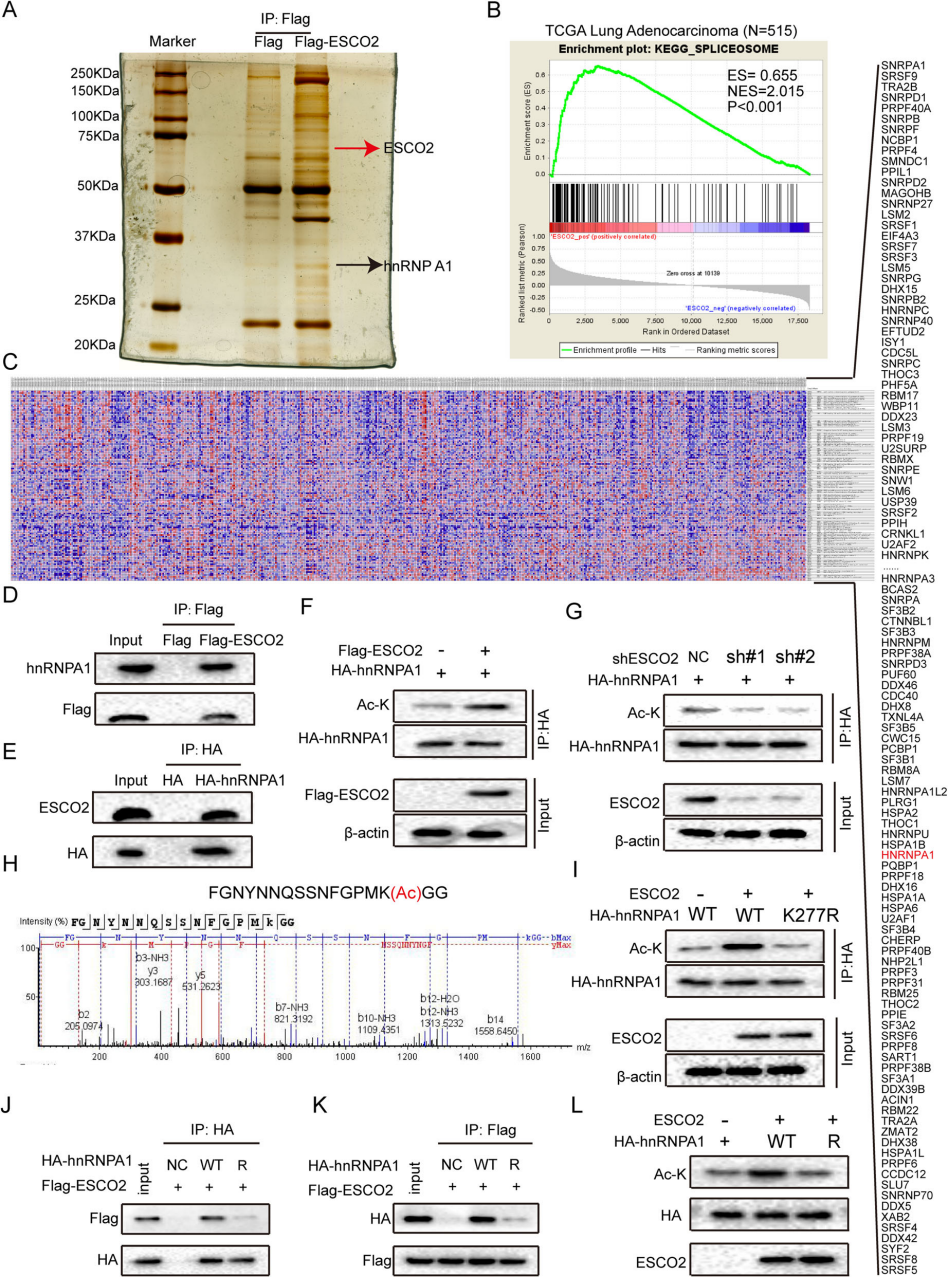
Interaction of ESCO2 with hnRNPA1 acetylates hnRNPA1 at K277. **a** *ESCO2-*FLAG vector was transfected into HEK293T cells, the ESCO2-FLAG complexes underwent Co-IP, and then ESCO2-binding proteins were identified by combined silver staining and MS. **b** Enrichment plot showing enrichment of spliceosome-related genes in the *ESCO2* high-expression group in TCGA LUAD cohort. **c** Heatmap showing the relative expression values for 126 spliceosome-related genes in TCGA LUAD cohort. **d** The *ESCO2-*FLAG vector was transfected into NCI-H1975 cells, the ESCO2-FLAG complexes underwent Co-IP, and then hnRNPA1 in the complexes was detected. **e** *hnRNPA1-*HAplasmids were transfected into NCI-H1975 cells, the hnRNPA1-HA complexes underwent Co-IP, and then ESCO2 in the complexes was detected. **f** NCI-H1975 cells were simultaneously transfected with *hnRNPA1-*HAand *ESCO2-*FLAGvectors, immunoprecipitated with anti-HA antibody, and then detection was performed using anti–ac-K antibody. **g** NCI-H1975 cells with *ESCO2* stable silencing were transfected with *hnRNPA1*-HA vector, immunoprecipitated by anti-HA antibody, and then detection was performed using anti–ac-K antibody. **h** The K277 acetylation site was identified by MS. **i** *hnRNPA1* WT or K277R mutant plasmids were co-transfected with *ESCO2-*FLAG into NCI-H1975 cells, and acetylation levels were detected using anti–ac-K antibody. **j** and **k** *hnRNPA1* WT or K277R mutant plasmids were co-transfected with *ESCO2-*FLAG into NCI-H1975 cells, anti-HA antibody was used for Co-IP, and ESCO2*-*FLAG was detected using anti-FLAG antibody (**j**); anti-FLAG antibody was used for Co-IP, and hnRNPA1 WT and K277R mutant constructs were detected using anti-HA antibody (**k**). Recombinant WT hnRNPA1 and its mutant K277R were incubated with recombinant ESCO2, and acetylation levels were detected using anti–ac-K antibody

The mechanism of ESCO2 action in LUAD progression was investigated using immunoprecipitation, silver staining, and MS, and 32ESCO2-binding proteins were identified (Fig. 4a and Table S2). The GSEA plot indicated that enrichment of the spliceosome-related genes was significantly correlated to high *ESCO2* mRNA expression levels in TCGA LUAD cohort (*p* < 0.001; Fig. 4b and c). The MS showed that hnRNPA1 had a high mass spectrum score (Table S2), and GSEA results and previous study showed that it is an important splicing regulator [27]. According to the results of the MS and GSEA, we found hnRNP A1 to be particularly interesting. To determine the interaction between ESCO2 and hnRNPA1, the *ESCO2-*FLAG vector was transfected into NCI-H1975 cells, the ESCO2-FLAG complexes underwent Co-IP, and then hnRNPA1 in the complexes was detected (Fig. 4d). NCI-H1975 cells were transfected with *hnRNPA1-*HAplasmids, the hnRNPA1-HA complexes underwent Co-IP, and then ESCO2 in the complexes was detected (Fig. 4e). As ESCO2 is an evolutionarily conserved cohesion acetyltransferase, we examined whether ESCO2 acetylates hnRNPA1 by co-transfecting NCI-H1975 cells with *hnRNPA1-*HAand *ESCO2-*FLAGvectors, followed by immunoprecipitation by anti-HA antibody, and detection using the pan-specific anti-acetylated lysineantibody. The hnRNPA1 acetylation levels were increased (Fig. 4f), suggesting that ESCO2 can acetylate hnRNPA1 protein. In addition, compared with the NC group, the hnRNPA1 acetylation levels in the shESCO2 group was decreased significantly (Fig. 4g). Co-IP confirmed that there was an interaction between ESCO2 and hnRNPA1 protein and that ESCO2 could acetylate hRNPA1 protein, but the acetylation site was not known. The acetylation site was identified by Co-IP combined with MS (Fig. 4g and Fig. S1), and was revealed to be on lysine (K) at site 277. To investigate whether the acetylation site is at K277, hnRNPA1 WT (wild-type) or K277R mutant plasmids were co-transfected with *ESCO2*-FLAG into NCI-H1975 cells, and acetylation levels were detected using anti–ac-K antibody. We found that the K277R mutant decreased ac-K expression (Fig. 4i). In addition, the K277R mutation influenced the interaction between ESCO2 and hnRNPA1 (Fig. 4j and k). To prove that ESCO2 specifically regulates hnRNPA1 acetylation, recombinant WT hnRNPA1 and its K277R mutant were incubated with recombinant ESCO2, and acetylation levels were detected using anti–ac-K antibody. The acetylation level significantly reduced in the K277R group (Fig. 4l). Collectively, our results indicate that ESCO2 binds to hnRNPA1 and regulates its acetylation. The acetylation site is at K277, which determines the interaction and acetylation between ESCO2 and hnRNPA1.

### ESCO2 acetylates hnRNPA1 at K277 to promote the aggressive phenotype of LUAD cells

**Fig. 5:**
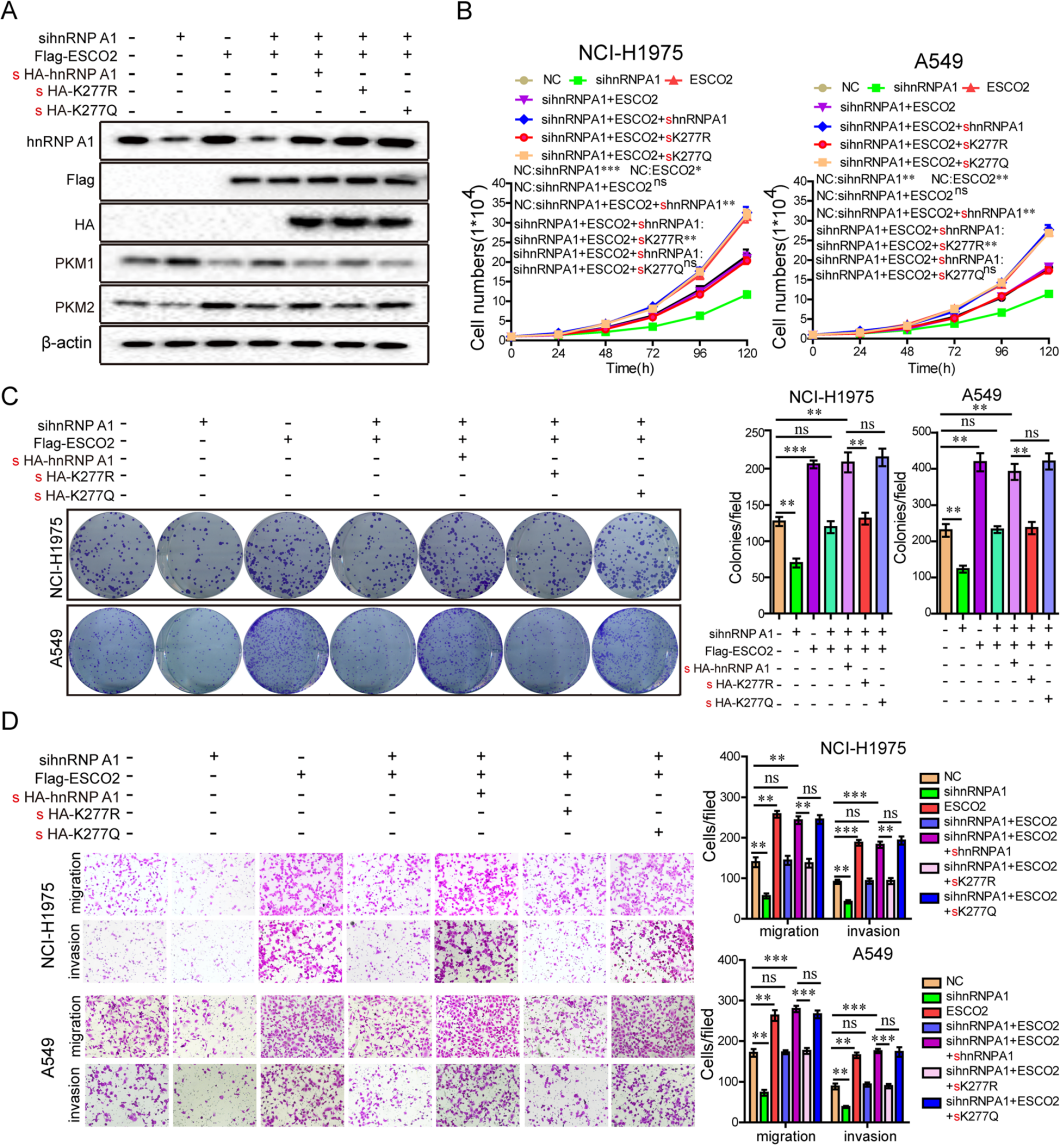
ESCO2 acetylates hnRNPA1 at K277, promoting the malignant phenotype of LUAD cells. After 36-h transfection of *hnRNPA1* siRNAs into LUAD cells, the cells were transfected with WT hnRNPA1 and its mutant K277R and K277Q plasmids, together with ESCO2 plasmids. Protein levels (**a**) and cell growth (**b**), migration (**c**), invasion (**c**), and colony formation (**d**) were detected. Data are the mean ± SD. *p < 0.05, **p < 0.01 or ***p < 0.001

Next, we explore whether ESCO2 promotes the malignant phenotype of LUAD cells by acetylating the K277 site of hnRNP A1.By knocking down the expression of hnRNPA1 by siRNA, the effect of the background expression of hnRNP A1 was eliminated. The *hnRNPA1* siRNAs (small interfering RNAs) together with ESCO2 plasmids were transfected into LUAD cells, and hnRNPA1 expression was restored using WT hnRNPA1 and the mutant K277R and K277Q plasmids (Fig. 5a). Compared with the NC group, silencing hnRNPA1 significantly suppressed LUAD cell growth, colony formation, migration, and invasion in the NCI-H1975 and A549 cells (Fig. 5b–d). Furthermore, silencing hnRNPA1 antagonized the enhancement of LUAD cell growth, colony formation, migration, and invasion induced by *ESCO2* overexpression, indicating that *ESCO2* promotes malignant progression through hnRNPA1 (Fig. 5b–d). Interestingly, when the WT hnRNPA1 vector or hnRNPA1 K277Q mutant restored hnRNPA1 expression in the LUAD cells, ESCO2 significantly promotes the malignant phenotype of LUAD cells, but not in the hnRNPA1 K277R mutant condition, as ESCO2 could not acetylate it (Fig. 5b–d). Collectively, these data show that *ESCO2* promotes LUAD cell proliferation and metastasis by acetylating the oncoprotein hnRNPA1 at K277.

### ESCO2 increases hnRNPA1 binding to the intronic sequences flanking exon 9 (EI9) of PKM mRNA by inhibiting hnRNPA1 nuclear export

**Fig. 6:**
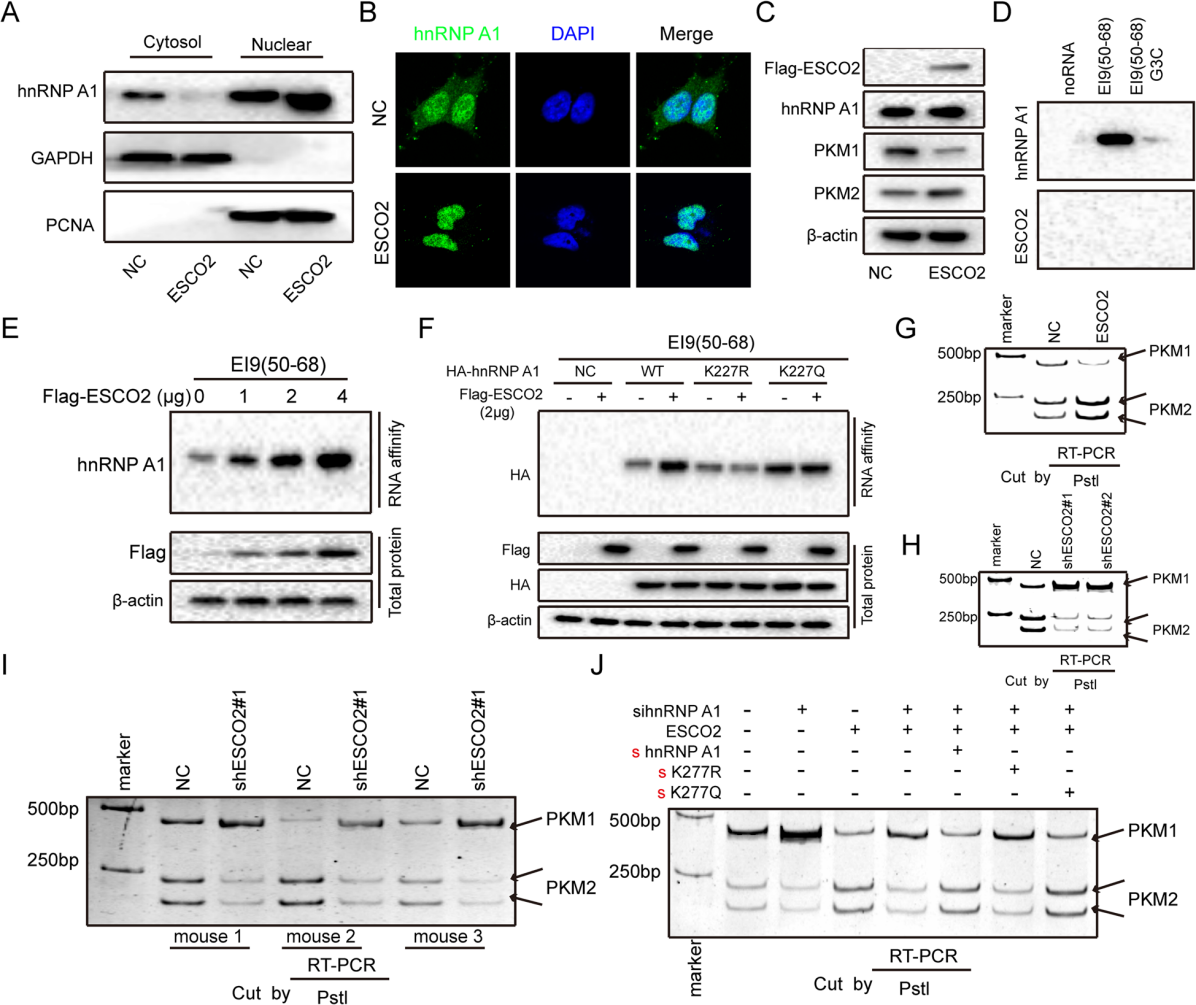
ESCO2 increases hnRNPA1 binding to the EI9 of *PKM* mRNA by inhibiting hnRNPA1 nuclear export, eventually inhibiting PKM1 isoform formation and inducing PKM2 isoform formation. **a**–**c** *ESCO2-*FLAGplasmid was transfected into NCI-H1975 cells; the nucleus and cytoplasm were separated, and then hnRNPA1 was detected using western blotting (**a**). Anti-hnRNPA1 antibody was used to detect the subcellular localization of hnRNPA1 (green) (**b**). Protein levels were detected using western blotting (**c**). **d** NCI-H1975 nuclear extracts were affinity-purified using biotin-labeled RNAs, and then ESCO2 and hnRNPA1 were detected. **e** *ESCO2*-FLAG vector was transfected into NCI-H1975 cells, RNA affinity purification was performed using biotin-labeled RNA EI9 (50–68), and then hnRNPA1 was detected. **f** WT hnRNPA1 and its mutant K277R and K277Q plasmids together with ESCO2 plasmids were co-transfected into NCI-H1975 cells, and RNA affinity purification was performed as in (**d**), and then hnRNPA1 was detected. **g**–**j** *PKM* splicing assay was performed by combining RT-PCR with *Pst*I. **g** *ESCO2*-FLAG vector was transfected into NCI-H1975 cells, and then PKM splicing assay was performed. **h** The *PKM* splicing of NCI-H1975 cells with *ESCO2* stable silencing was detected. **i** *PKM* splicing was performed in mouse xenograft tumors (*n* = 3). **j** After 36-h transfection of hnRNPA1 siRNAs into NCI-H1975 cells, the cells were transfected with WT hnRNPA1 and its mutant K277R and K277Q plasmids together with ESCO2 plasmids, and then the *PKM* splicing assay was performed

The K277 site of hnRNPA1 is located in the M9 domain, which mediates hnRNPA1 nuclear transport [28, 29]. Therefore, we investigated if hnRNPA1 acetylation by ESCO2 affects its nuclear localization. The *ESCO2-*FLAGplasmid was transfected into NCI-H1975 cells; the nucleus and cytoplasm were separated. hnRNPA1 expression in the *ESCO2* overexpression cells was upregulated in the nucleus, and was downregulated in the cytosol (Fig. 6a). Immunofluorescence staining indicated that *ESCO2* overexpression retained hnRNPA1 in the nucleus (Fig. 6b). *ESCO2* overexpression upregulated PKM2 protein expression levels and downregulated PKM1 protein expression levels, but did not change hnRNPA1 expression levels (Fig. 6c).

hnRNPA1 restricts the inclusion of PKM exon 9, promoting PKM2 formation and inhibiting PKM1 formation, by binding to the UAGGGC sequences of exon 9 [23, 30]. RNA pull-down experiments showed that hnRNPA1 can strongly bind to the PKM EI9 (50–68) sequence, and when the G3 nucleotide of EI9 (50–68) is mutated to C, its ability to bind to hnRNPA1 is significantly reduced (Fig. 6d). At the same time, ESCO2 did not directly bind to the EI9 (50–68) sequence (Fig. 6d). Moreover, ESCO2 increased hnRNPA1 and PKM EI9 (50–68) binding in a dose-dependent manner in the nucleus (Fig. 6e). To investigate whether ESCO2 increases hnRNPA1 to PKM EI9 (50–68) binding by acetylating hnRNPA1, we transfected *hnRNPA1* siRNAs into NCI-H1975 cells for 36 h, and then co-transfected the cells with WT hnRNPA1 and its mutant K277R and K277Q plasmids together with ESCO2 plasmids RNA pull-down using biotin-labeled EI9 (50–68) RNA showed that that ESCO2 promoted the binding of WT hnRNPA1 to PKM EI9 (50–68), but not that of the K277R mutant. In addition, compared to the WT hnRNPA1, the K277Q mutant had higher affinity with PKM EI9 (50–68), and *ESCO2* overexpression did not increase hnRNPA1 binding to PKM EI9 (50–68) (Fig. 6f). Next, we used RT-PCR and *Pst*I restriction digestion to investigate whether ESCO2 regulates alternative splicing of *PKM* pre-mRNA. *ESCO2* overexpression decreased *PKM1* isoform mRNA levels and increased that of the *PKM2* isoform (Fig. 6g). Silencing *ESCO2* decreased *PKM2* isoform mRNA levels and increased that of the *PKM1* isoform (Fig. 6h); the same results were obtained for *PKM* splicing in the mouse xenograft tumors (*n* = 3) (Fig. 6i). Compared with the NC group, silencing hnRNPA1 suppressed *PKM2* isoform mRNA levels and increased that of the *PKM1* isoform, and silencing hnRNPA1 antagonized the splicing change of *PKM* pre-mRNA induced by *ESCO2* overexpression (Fig. 6j). Furthermore, when the WT hnRNPA1 vector or hnRNPA1 K277Q mutant restored hnRNPA1 expression in the LUAD cells, ESCO2 could decrease *PKM1* isoform mRNA levels and increased that of the *PKM2* isoform, but not in the hnRNPA1 K277R mutant condition. Collectively, these data show that ESCO2 increases hnRNPA1 binding to the EI9 of *PKM* mRNA by inhibiting hnRNPA1 nuclear translocation, eventually inhibiting PKM1 isoform formation and inducing PKM2 isoform formation.

#### ESCO2 promotes aerobic glycolysis of LUAD cells by increasing PKM2 expression and decreasing PKM1 expression

**Fig. 7:**
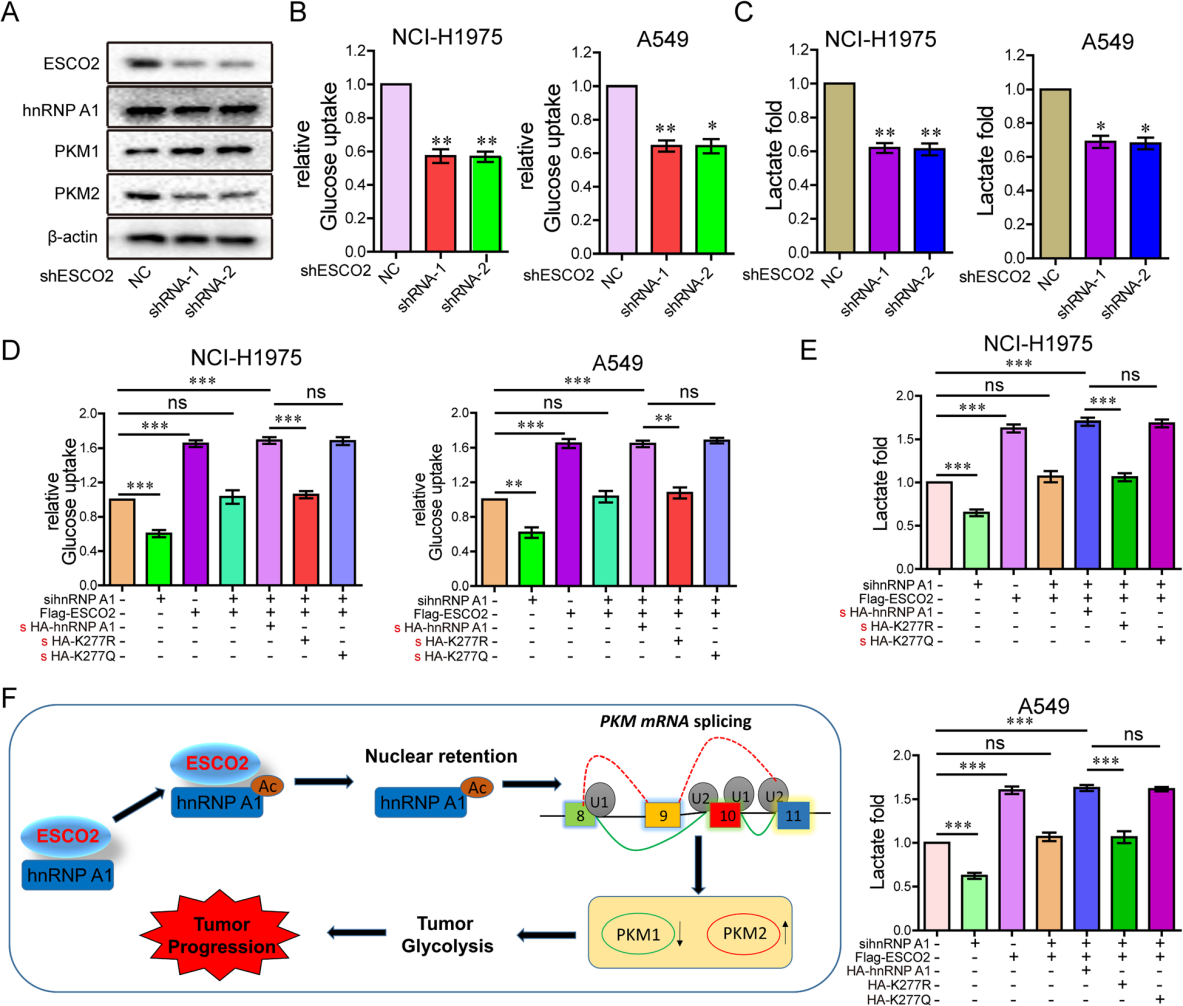
ESCO2 promotes aerobic glycolysis of LUAD cells by increasing PKM2 expression and decreasing PKM1 expression. **a** Protein levels were detected in NCI-H1975 cells with *ESCO2* stable silencing. **b** and **c** Glucose uptake (**b**) and lactate production (**c**) were measured in LUAD cells with *ESCO2* stable silencing. **d** and **e** After 36-h transfection with hnRNPA1 siRNAs into LUAD cells, the cells were transfected with WT hnRNPA1 and its mutant K277R and K277Q plasmids together with ESCO2 plasmids, and then glucose uptake (**d**) and lactate production (**e**) were examined. **f** The working model of ESCO2 promotes aerobic glycolysis and drives tumorigenesis by acetylating hnRNPA1

The PKM2 isozyme is a key promoter of the Warburg effect in tumors, which is characterized by increased glucose uptake and lactic acid production [31]. PKM protein levels were detected in NCI-H1975 cells with *ESCO2* stable silencing, and showed that PKM1 expression was obviously increased, while PKM2 expression was obviously decreased (Fig. 7a). Silencing *ESCO2* significantly decreased glucose uptake and lactate production in the LUAD cells (Fig. 7b and c). In addition, *ESCO2* overexpression significantly increased glucose uptake and lactate production in the LUAD cells (Fig. S2). Compared with the NC group, silencing hnRNPA1 also significantly decreased glucose uptake and lactate production, and antagonized the glucose uptake and lactate production promoted by *ESCO2* overexpression (Fig. 7d and e). Furthermore, when the WT hnRNPA1 vector or hnRNPA1 K277Q mutant restored hnRNPA1 expression in the LUAD cells, ESCO2 significantly promotes glucose uptake and lactate production, but not in the hnRNPA1 K277R mutant condition (Fig. 7d and e). The working model of ESCO2 promoted aerobic glycolysis and drove malignant progression by acetylating hnRNPA1 (Fig. 7f).

## Discussion

In the present study, literature and database analyses showed that ESCO2 has a significant correlation with malignant progression of LUAD. Here, comparison of tumor-adjacent normal lung tissue via analysis of the Oncomine, GEO and TCGA datasets showed that ESCO2 mRNA and protein expression levels were upregulated in LUAD tissues. GSEA showed that the cell cycle signatures and DNA replication signatures had a significant positive correlation with *ESCO2* mRNA expression levels in TCGA LUAD cohort. Our results show that The *ESCO2* overexpression NCI-H1975 and A549 cells had greater cell growth and greater invasive, migration, and colony-formation ability, while silencing *ESCO2* had the opposite effect. Furthermore, *ESCO2* stable silencing decreased the tumor growth and pulmonary metastasis of the NCI-H1975 cells in vivo. Chen et al. discovered that *ESCO2* knockdown dramatically inhibited cell proliferation and induced apoptosis in human gastric cancer cells, and suppressed tumor xenograft development in vivo [19]. In addition, significantly high *ESCO2* expression was found in renal cell carcinoma tissue and cell lines, promoting cell aggressive behaviors and inducing poor prognosis [15]. Therefore, together with other research data, our data indicate that ESCO2 plays a key role in the proliferation and metastasis of many types of human cancer.

Furthermore, we found that ESCO2 could interact with hnRNPA1 and acetylates hnRNPA1 at K277. hnRNPA1 regulates alternative splicing of interferon regulatory factor 3 and affects immunomodulatory functions in human non–small cell lung cancer cells [32]. hnRNPA1 plays a crucial role in regulating cell proliferation, invasiveness, metabolism, and immortalization in multiple tumors such as hepatocellular carcinoma, prostate cancer, and oral squamous cell cancer [33–35]. In the present study, silencing hnRNPA1 antagonized the enhancement of LUAD cell growth, colony formation, migration, and invasion induced by*ESCO2* overexpression. The data suggest that the ESCO2 and hnRNPA1 interaction promotes the malignant progression of LUAD.

Here, we discovered a novel acetylated substrate, hnRNPA1, of the acetyltransferase ESCO2. Furthermore, we found that ESCO2 acetylates hnRNPA1 at K277 and inhibits the nuclear export of hnRNPA1. In addition, hnRNPA1 mediated the regulation of *PKM* splicing by blocking the binding of the arginine residues in the RGG motif of hnRNPA1 to the PKM EI9, ensuring the formation of PKM2 and suppressing glucose metabolism reprogramming [26]. We discovered that ESCO2 increased hnRNPA1 binding to the EI9 of *PKM* mRNA by inhibiting hnRNPA1 nuclear translocation, leading to increased PKM2 expression and decreased PKM1 expression.

We also elucidated the function and mechanism of ESCO2 in glucose metabolism in LUAD cells. ESCO2 promoted aerobic glycolysis of LUAD cells by increasing PKM2 expression and decreasing PKM1 expression. PKM is a glycolytic enzyme that catalyzes the final step in glycolysis, and exists in two different forms: PKM1 and PKM2. PKM1 is distributed in high energy–demand organs, such as brain and muscle. *PKM2* is believed to be one of the most important genes in cancer-specific energy metabolism, known as the Warburg effect [36]. Most cancer cells such as that of colon cancer, bladder cancer, and pancreatic cancer express PKM2 dominantly to maintain a glycolysis-dominant energy metabolism [24, 37–39]. PKM2 reduces the glucose levels for intracellular utilization, in particular citrate production, thereby increasing the α-ketoglutarate/citrate ratio to promote the generation of glutamine-derived acetyl-coenzyme A through the reductive pathway. In addition, reductive glutamine metabolism promotes cell proliferation under hypoxia conditions and supports in vivo tumor growth [40]. Therefore, we have found and proven that ESCO2 is an important functional molecule that promotes metabolic reprogramming of LUAD. The ESCO2-cohesin complex links DNA molecules and plays important roles in the gene transcription of eukaryotic genomes. Sadia Rahman and their colleagues have found that Esco2 binding sites are enriched for CTCF and REST/NRSF transcription factor motifs [41]. In addition, ESCO2 can regulate transcription of neuron-specific genes in other tissues [42, 43]. These studies show that ESCO2 is closely related to RNA transcription regulation. Whether ESCO2 promotes the malignant progression of lung adenocarcinoma by regulating gene transcription and its specific regulatory mechanisms, this will be the focus of our further research.

## Conclusion

In conclusion, patients with LUAD with high ESCO2 mRNA and protein expression levels have lower OS and recurrence-free survival, as compared with patients with low *ESCO2* expression levels. A novel role for ESCO2 in LUAD tumorigenesis has been elucidated, that is, *ESCO2* upregulation promotes cell growth, proliferation, colony formation, and cell cycle progression. Moreover, ESCO2 can interact with hnRNPA1 and acetylates it at K277, retaining it in the nucleus. In addition, ESCO2 promotes hnRNPA1 binding to the EI9 of *PKM* mRNA by inhibiting hnRNPA1 nuclear translocation. Furthermore, ESCO2 promotes aerobic glycolysis of LUAD cells by increasing PKM2 expression and decreasing PKM1 expression. Therefore, ESCO2 may serve as a new therapeutic target for LUAD.

## Supplementary Information

**Additional file 1: Supplementary Figure 1.** (A) *hnRNPA1-HA* vector was transfected into HEK293T cells, the *hnRNPA1-HA* complexes underwent Co-IP, and then protein modification of hnRNPA1 was identified by Coomassie blue staining with MS.

**Additional file 2: Supplementary Figure 2.** (A-B) *ESCO2*-FLAG vector was transfected into NCI-H1975 cells, and then Glucose uptake (A) and lactate production (B) were measured.DD.

**Additional file 3: Supplementary Table S1.** The antibodies, primers , oligonucleotides and shRNAs used in this study are shown.

**Additional file 4: Supplementary Table S2.**

## Data Availability

All data generated or analyzed during this study are included in thispublished article.

## Abbreviations

ESCO2: Establishment of cohesion 1 homolog 2
hnRNPA1: Heterogeneous nuclear ribonucleoprotein A1
LUAD: Lung adenocarcinoma
Co-IP: Coimmunoprecipitation
MS: Mass spectrometry
GSEA: Gene set enrichment analysis
