# Supplementary figures and images for: ESCO2 promotes lung adenocarcinoma progression by regulating hnRNPA1 acetylation
Source: J Exp Clin Cancer Res. 2021 Feb 11;40:64. doi: 10.1186/s13046-021-01858-1 (PMC7876794; doi:10.1186/s13046-021-01858-1)

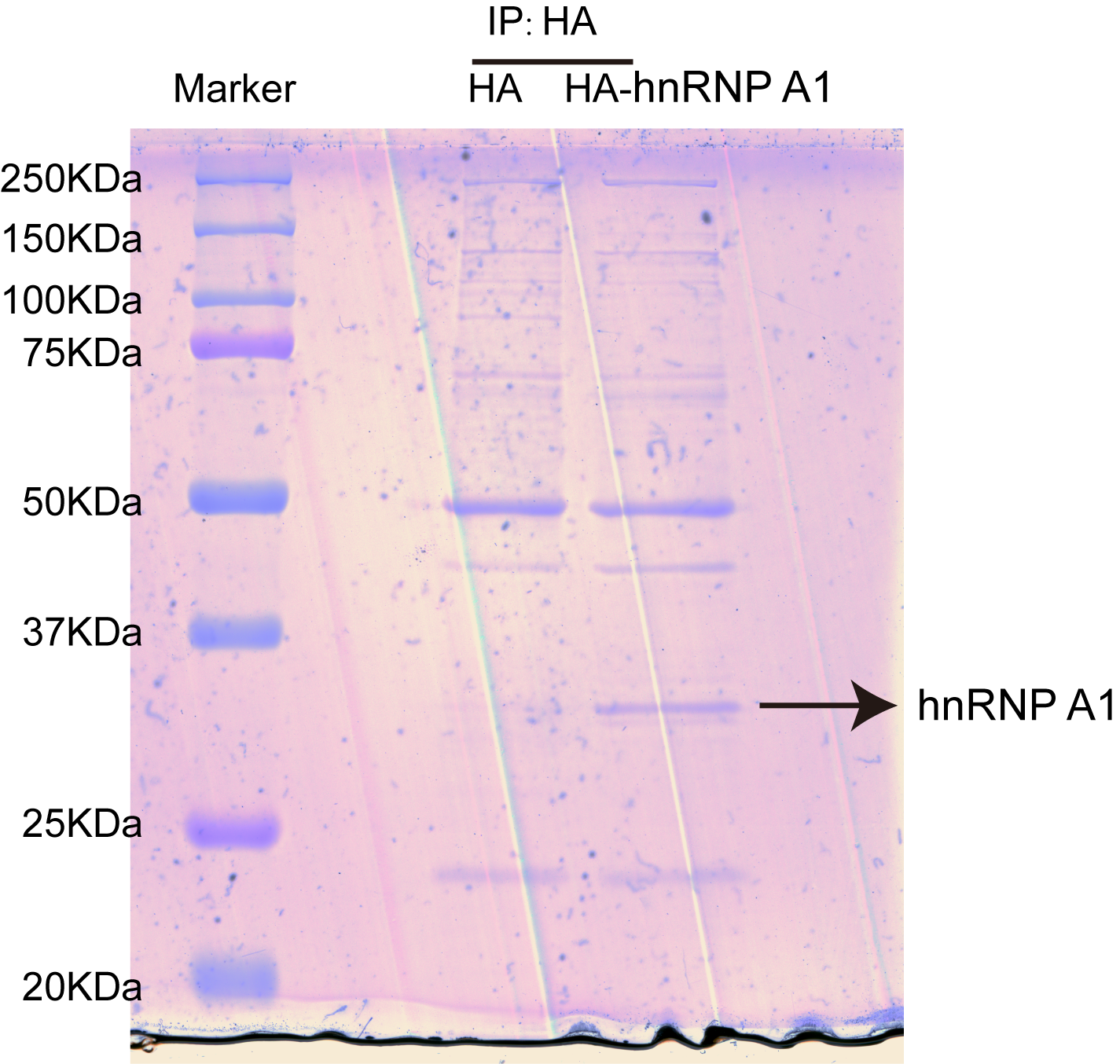

Supplement: Supplementary file 1 — Additional file 1: Supplementary Figure 1. (A) hnRNPA1-HA vector was transfected into HEK293T cells, the hnRNPA1-HA complexes underwent Co-IP, and then protein modification of hnRNPA1 was identified by Coomassie blue staining with MS. [file 13046_2021_1858_MOESM1_ESM.tif]

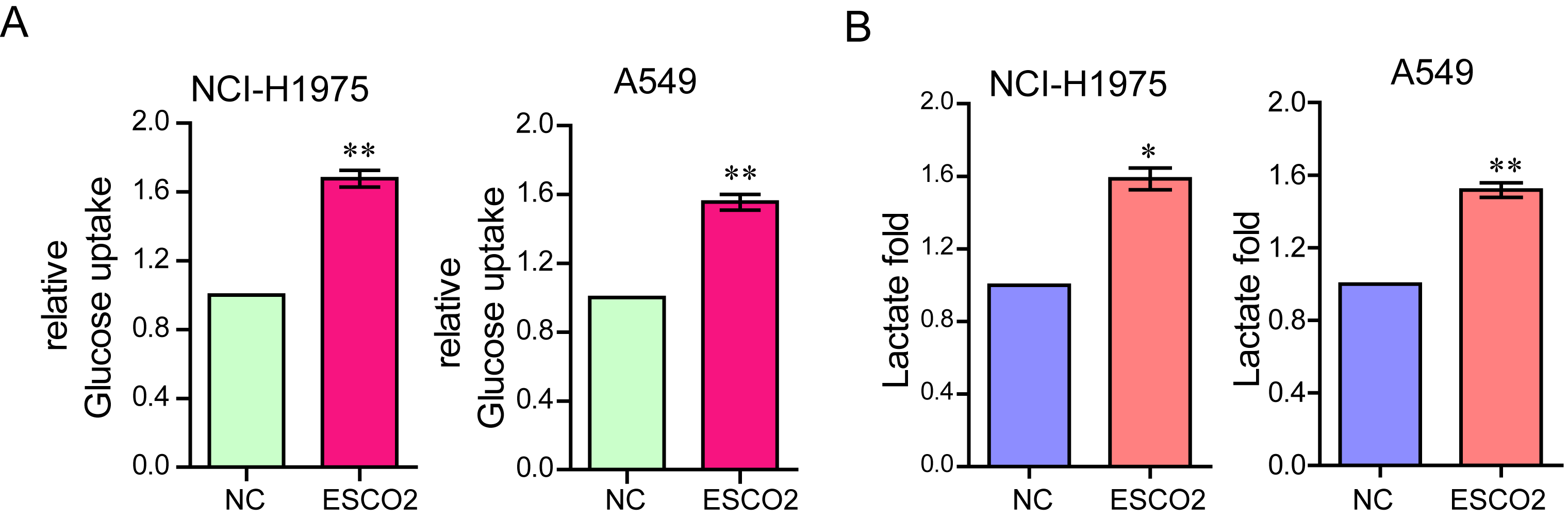

Supplement: Supplementary file 2 — Additional file 2: Supplementary Figure 2. (A-B) ESCO2-FLAG vector was transfected into NCI-H1975 cells, and then Glucose uptake (A) and lactate production (B) were measured.DD. [file 13046_2021_1858_MOESM2_ESM.tif]
