# Supplementary material for: ESCO2 promotes lung adenocarcinoma progression by regulating hnRNPA1 acetylation
Source: J Exp Clin Cancer Res. 2021 Feb 11;40:64. doi: 10.1186/s13046-021-01858-1 (PMC7876794; doi:10.1186/s13046-021-01858-1)
Supplement: Supplementary file 3 — Additional file 3: Supplementary Table S1. The antibodies, primers , oligonucleotides and shRNAs used in this study are shown. [file 13046_2021_1858_MOESM3_ESM.docx]

| **Table S1. The antibodies, primers ,oligonucleotides and shRNAs used in this study are shown.** | | |
| --- | --- | --- |
| **Antibodies** | | |
| Rabbit Polyclonal anti-ESCO2 | Biorbyt | Cat# orb183433 |
| Rabbit Polyclonal anti- hnRNP A1 | Proteintech | Cat# 11176-1-AP |
| Rabbit Polyclonal anti-Flag | abcam | Cat# ab1162 |
| Rabbit Polyclonal anti-HA | abcam | Cat# ab9110 |
| Rabbit Polyclonal Acetylated-Lysine | Cell Signaling Technology | Cat# 9441 |
| Rabbit Polyclonal anti-PKM1 | Proteintech | Cat# 15821-1-AP |
| Rabbit Polyclonal anti-PKM2 | Proteintech | Cat#15822-1-AP |
| Mouse monoclonal anti-β-actin | Santa Cruz | Cat# sc-8432 |
| Rabbit Polyclonal anti-GAPDH | Proteintech | Cat# 10494-1-AP |
| **Primers name** |  |  |
| ESCO2  (Q-PCR) | Forward | TCATCATCGACGCTGGTCA |
|  | Reverse | AAAACTCTGCTACTACACGTT |
| GAPDH  (Q-PCR) | Forward | CCTCTGACTTCAACAGCGACACC |
|  | Reverse | ACCACCCTGTTGCTGTAGCCAA |
| PKM  (RT-PCR) | Forward | CTGAAGGCAGTGATGTGGCC |
|  | Reverse | ACCCGGAGGTCCACGTCCTC |
| ESCO2-Flag | Forward | ATGGCAGCTCTTACTCCAAGGA |
|  | Reverse | TTACTTGTCATCGTCGTCCTTGTAGTCACTATTAAAATTATATACGAGGAAATTAGG |
| hnRNP A1-HA | Forward | AGATACGTTCGTCAGCTTGCTCCT |
|  | Reverse | TTAAGCGTAATCTGGAACATCGTATGGGTATGTAACCTGTAGCT |
| hnRNPA1-  K277R-HA | Forward | acccatgaagggaggaaattttGGAGGCAGAAGCTCTGGCC |
|  | Reverse | ttcctcccttcatgggtccaaaATTTGAAGACTGATTGTTGTAATTCCC |
| hnRNPA1-  K277Q-HA | Forward | ccatgcagggaggaaattttGGAGGCAGAAGCTCTGGCC |
|  | Reverse | atttcctccctgcatgggtccaaaATTTGAAGACTGATTGTTGTAATTCCC |
| **Oligonucleotides** | | |
| Control shRNA | sense: 5‘-CCGGGCAGCTTTTTTG-3‘ | |
| ESCO2 shRNA#1 | sense:5‘-CCGGGCACCTTACTTGTTCTGAGATCTCGAGATCTCAGAACAAGTAAGGTGCTTTTTTG-3‘ | |
| ESCO2 shRNA#2 | sense:5‘-CCGGGCAAGTCTTGTGGTATGATATCTCGAGATATCATACCACAAGACTTGCTTTTTTG-3‘ | |
| siNC: | sense: 5‘-GCACAAGCUGGAGUACAACUACATT-3‘ | |
| sihnRNP A1 | sense: 5‘- GUGUAGUUGAACUGAUAGUTT -3‘, | |
| EI9(50-68) | Biotin-AGGUAGGGCCCUAAGGGCA | |
| EI9(50-68, G3C) | Biotin-AGGUACGGCCCUAAGGGCA | |
